# Artificial Intelligence–Powered Answers to Questions in Genomic Medicine

**DOI:** 10.1007/s40670-025-02444-2

**Published:** 2025-07-04

**Authors:** Mary Kate Worden, Allison Cruise, Johanna M.B. Craig, Eli S. Williams

**Affiliations:** 1 Office of Medical Education, Center for Medical Education Research and Scholarly Innovation, University of Virginia School of Medicine, Charlottesville, VA USA; 2 Health Sciences Library, University of Virginia School of Medicine, Charlottesville, VA USA; 3 Medical Education Technology, University of Virginia School of Medicine, Charlottesville, VA USA; 4 Department of Pathology, University of Virginia School of Medicine, Charlottesville, VA USA

**Keywords:** Instructional design, Artificial intelligence (AI), Chat GPT, Critical appraisal, Genomic medicine

## Abstract

We asked first-year medical students to critique ChatGPT-generated responses to questions in genomic medicine. Students compared AI outputs to biomedical literature, highlighting AI’s strengths and limitations. The exercise fosters critical thinking about AI in healthcare, in alignment with recommendations for integrating AI into medical education.

Artificial intelligence–powered chatbots like ChatGPT have shown potential in a variety of medical contexts, including research, diagnosis, and patient monitoring, but are also prone to error and bias. To help incoming first-year medical students recognize the strengths and limitations of using artificial intelligence (AI) as a source for biomedical information, we asked them to critique ChatGPT-authored responses to questions in genomic medicine by comparing the output of ChatGPT to information students retrieved from the biomedical literature. As a pre-class assignment, students watched a video produced by *The New England Journal of Medicine* that explains how large language models like ChatGPT work [1]. 

The illustration in Fig. 1 shows the sequence of the exercise. A geneticist elicited ChatGPT responses to ten different prompts about genetic disease. Each prompt consisted of a clinical vignette about a patient with a high suspicion of genetic disease and four clinical questions about the patient. None of the diseases had been covered yet in the curriculum. Each student (*n* = 155) was assigned to review one prompt with the corresponding response from ChatGPT and to critique the accuracy, clarity, and completeness of the AI response in 300–500 words. Students had two weeks to write their essay and were required to cite 4–5 primary or secondary sources from the biomedical literature in support of their critique. This requirement encouraged incoming students to practice skills for effectively searching the literature that were taught earlier in orientation.

**Fig. 1:**
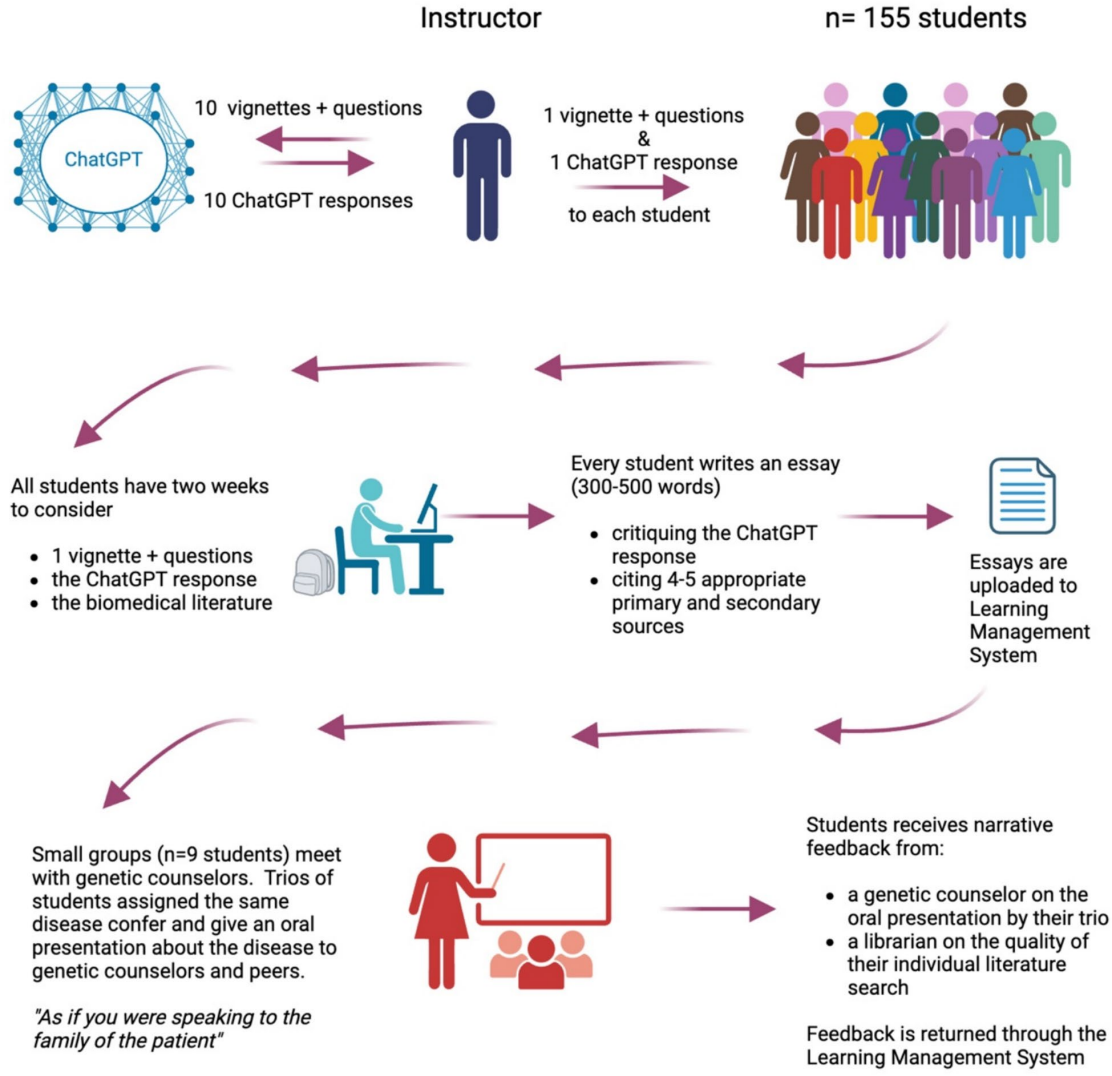
Flowchart illustrating the sequence of elements in the exercise “Artificial Intelligence–Powered Answers to Questions in Genomic Medicine” (see text). Created in BioRender. Education, M. (2025) https://BioRender.com/l24e527

On the day students submitted their essays and supporting references, they also met in small groups (*n* = 9 students) for 90 min with a genetic counselor. Within each small group, three students had received the same assignment; these trios compared their critiques of AI and collaborated to deliver a brief oral presentation about the disease “as if they were speaking to the family of the patient.” Afterwards, the small group discussed the promise and perils of using AI in healthcare, and the genetic counselors gave each trio narrative feedback on the quality and accuracy of their oral presentation. A health sciences librarian subsequently provided narrative feedback to each student on the quality of the references they had cited in their essays. Correct “answers” to each set of 10 prompts were not distributed to students; however, instructors in subsequent pre-clerkship courses taught each of the genetic diseases. 

In their written essays, students reported that ChatGPT generally provides a succinct, useful summary for a layperson who wants more information about a disease. However, students also referenced primary and secondary biomedical literature sources to argue that AI outputs lack details important for diagnosis and management. 

On course evaluations, student commentary about this exercise was generally positive.

- *Student 1: *
- *I didn't realize how many people actually use ChatGPT to learn more about their symptoms. It made me realize that this is a tool that I would need to work with and educate myself, staff and my patients on how to appropriately use. *
- *Student 2: *
- *It's great to tackle the AI"issue"head on and within a medical context. This session made a lot of things clear about the current limits of large language models, shed some light on the directions where this technology could be utilized, and made it apparent that these tools will be present in the medical profession in the years to come. *
- *Student 3: *
- *This was a valuable experience in understanding the professional implications of this novel technology and was helpful both in learning the genomics content and how to think critically about how this information relates to us as physicians and to our future patients.*

However, other students reported that the small group discussion felt superficial, or that writing the essay seemed superfluous, or that focusing on ChatGPT output was unhelpful.

- *Student 1:*
- * It's an interesting topic, but I've had deeper conversations regarding AI and medicine in my day-to-day conversations with other students. *
- *Student 2: *
- *This session was interesting because it made me think critically about AI, but I think that the essay requirement was not necessary. *
- *Student 3: *
- *I think the discussion of AI is incredibly important; however, using Chat-GPT as the example was unhelpful. It's not trained to be a medical assistant, so of course it does a poor job of answering clinical questions.*

Five elements of this curricular exercise are key to its success. First, the timing of this exercise is critical. Scheduling it within the first month of the medical curriculum ensures that it is the first curricular exercise focused on the topic of AI in medical education, that it precedes any formal instruction on genetic diseases, and that it provides early encouragement for incoming students to familiarize themselves with resources available through the health science library. Second, assigning the NEJM video provides students with a succinct and engaging introduction to large language models by comparing LLMs to flash cards, an analogy that resonates with medical students. Third, limiting the focus to ten known sets of ChatGPT output allows instructors to anticipate the students’ critiques, whereas allowing students to design their own prompts would have generated over 150 different AI outputs. Fourth, incorporating small group discussions allows students to compare their critiques of AI output with peers. Fifth, providing students with individualized feedback on the references they cite helps incoming students recognize the strengths and weaknesses of their strategy for searching the biomedical literature.

Safely implementing AI in the care of patients and populations requires that health professionals employ critical appraisal of its power and limitations [2]. Requiring medical students to do the deep cognitive work of critically appraising AI-powered answers to questions in genomic medicine helps nurture those skills. Moreover, including an opportunity for small group discussions of AI-powered outputs aligns with several of the recommendations from the International Advisory Committee on Artificial Intelligence for integrating AI into undergraduate medical education, including that learners develop self-awareness about their knowledge, skills, and attitudes towards AI, maintain critical awareness regarding potential limitations in AI tools’ accuracy and completeness, and compare and contrast these thoughts and feelings with others [3].

## Data Availability

The data in this study is available upon request made to the corresponding author, provided this request complies with federal privacy guidelines regarding educational data.

## Declarations

This is an observational study. The Institutional Review Board reviewed and exempted this project from further review (Protocol #7437).
